# A novel benzamine lead compound of histone deacetylase inhibitor ZINC24469384 can suppresses HepG2 cells proliferation by upregulating NR1H4

**DOI:** 10.1038/s41598-019-39487-6

**Published:** 2019-02-20

**Authors:** Qiuhang Song, Mingyue Li, Cong Fan, Yucui Liu, Lihua Zheng, Yongli Bao, Luguo Sun, Chunlei Yu, Zhenbo Song, Ying Sun, Guannan Wang, Yanxin Huang, Yuxin Li

**Affiliations:** 1 National Engineering Laboratory for Druggable Gene and Protein Screening, Northeast Normal University, Changchun, 130024 China; 2 Research Center of Agriculture and Medicine gene Engineering of Ministry of Education, Northeast Normal University, Changchun, 130024 China

## Abstract

Histone deacetylases (HDACs) can enzymatically transferred acetyl functional group from protein or lysine residues of histone, so they can regulate the expression of lots of genes. Now HDACs are used as drug targets and many HDAC inhibitors (HDACis) were approved for cancer therapy or in clinical trials. However, the physiological mechanisms and regulatory processes of HDACi anti-cancer effects are largely unexplored and uncompleted. Here we use the virtual screening workflow obtained 25 hit compounds and ZINC24469384 can significantly inhibit HDAC activity while arrest cell cycle at G1/S phase and significantly induced HepG2 cell apoptosis, time-course RNA-seq demonstrate that HepG2 cells transcriptionally respond to ZINC24469384. Pathway analysis of DEGs and DASGs reveal that NR1H4 may play an important role in ZINC24469384-induced anti-proliferation effect and is dramatically alleviated by down-regulating the SOCS2 expression and promoting STAT3 phosphorylation in knockdown NR1H4 HepG2 cells. Analysis based on TCGA database indicated that NR1H4 and SOCS2 were downregulated in liver cancer, this suggest NR1H4 and SOCS2 may play an important role in tumorigenesis. These results indicated that ZINC24469384 is a novel benzamine lead compound of HDACi and provides a novel mechanism for HDACi to inhibit cancer.

## Introduction

Histone deacetylases (HDACs) and histone acetyl transferases (HATs) have been indicated that can regulate the acetyl functional group in histones and large numbers of non-histone proteins^1^. HDACs and HATs play an essential role in gene regulation. HDACs were involved in condensing chromatin so can downregulating many genes expression, while HATs can removes the positive charge on the histones, so the chromatin can transform to a more open structures and active the transcription. In recently study global hypoacetylation of histone is also correlated with numerous specific processes like the occurrence and development of tumor, with the features of uncontrolled cell growth, proliferation and so on^1,2^. Now, 11 classical human HDACs have been identified and grouped into three Classes based on their sequence homology to yeast orthologues Rpd3, Hdal and Sir2, respectively^3^. They are all Zn^2+^ dependent enzymes harboring a binding pocket with a Zn^2+^ chelating compounds^4^. Due to different functions of each HDAC in the cells, HDACi can induce lots of cellular changes in cancer cells and has been shown to reduce many pathways associate with tumor genesis. Previous studies reported that HDACi were able to modulate a variety of cellular functions including cell cycle arrest, inactivation of tumor suppressor genes, differentiation, inhibition of angiogenesis and induction of apoptosis^5^. So HDACis are playing increasingly key role in expanding field of anticancer drugs^3^. To date, five HDACis have been used for cancer therapy. Vorinostat, Romidepsin, Belinostat, Panobinostat and Chidamide are used for treatment of cutaneous T-cell lymphoa, and peripheral T-cell lymphoma and multiple myeloma. Now almost 15 new HDACis are in different stage of clinical trial and a number of candidates are under preclinical investigation in various malignancies which indicate the rapid development of the field of HDACi^6^. Although various HDACis are currently used to treat cancer in clinical, but toxicities including thrombocytopaenia and fatigue were also additionally observed^7^. So develop new HDACi is still urgently needed.

At present, HDAC inhibitors were developed in the absence of complete understanding of mechanism. And we also unclear that whether different structures of HDACis have the similar mechanisms of anti-tumor effects in different cell types^8^. Therefore, understanding the mechanisms of HDACi-induced cancer cell viability could provide new insights in cancer treatment. We all know that the apoptosis induced by HDACi is mediated by extrinsic pathway and/or mitochondrial pathway. The expression of TNF receptors and their ligands were upregulated after HDACi treated^9^. There also have been many independent studies strongly supporting the role for HDACi-mediated apoptosis in intrinsic pathway^6,8–10^. For example, HDACi could upregulate pro-apoptotic associated proteins, such as Bim, Bmf and Bax, HDACi could also downregulate anti-apoptotic proteins, like Bcl-2 and Bcl-XL^6,11^. It was also found that HDACi could not induced cell death in Bcl-2 overexpressed cells while down expression of Bcl-2 can increase the sensitivity of cells to HDACi^10^. Moreover, almost all HDACi studied to date, can induce cell cycle arrest at G1/S phase, that often related to induce the expression of cyclin-dependent kinase inhibitor (p21)^12^. While the upregulated expression of p21 might not the only reason for the cell cycle arrest, as many cyclin genes like Cyclin A, Cyclin B and Cyclin D can also induce cell cycle arrest in cancer^13^. There also have other potential mechanisms that can induce cell cycle arrest, like upregulated the expression of GADD45 and TGFβ receptor signaling associated genes^14,15^. Moreover, HDACi can also inhibits JAK/STAT signaling pathway avoid cancer cells from survival^16^.

Even though HDACi paly an important role in induce cancer cell apoptosis, antiangiogenesis and cell cycle arrest, while, the mechanism of the anti-cancer effects of ZINC24469384 remain to be fully elucidated.

In our study, we use the virtual screening workflow described in our previous study to select new HDACi^17^, we selected out 25 hit compounds for activity test and obtained a promising benzamides lead compounds - ZINC24469384. It can significantly inhibited the activity of HDACs, it can also selectively induce apoptosis and inhibit proliferation of live cancer cells. To know the molecular mechanisms underlying ZINC24469384 suppressed viability of HepG2 cells, we used time-course RNA-seq to identify differentially expressed genes/alternative spliced genes (DEGs/DASGs), and subsequently identified that ZINC24469384 induced genes were cross-talked. And active of FXR/RXR (NR1H4) may play an important role in ZINC24469384-regulated anti-proliferation effect based on the functional analysis using IPA. Experimental results conformed that downregulating NR1H4 in HepG2 cell can dramatically alleviate ZINC24469384-induced anti-proliferative effect. Previous study demonstrated that NR1H4 plays a key role in regulate the metabolism of glucose and liver regeneration^18^. In our study, we also found that NR1H4 play a key role in inhibiting STAT3 activation induced by ZINC24469384 through upregulating SOCS2 expression which leads to transcription of various genes related to apoptosis and cell cycle arrest. The findings of our results advance our knowledge in the mechanism of HDACi-induced tumor cell apoptosis and suggest that ZINC24469384 is a novel benzamine lead compound of histone deacetylase inhibitor, may has potential therapeutic effect in treating human hepatoma.

## Results

### Pharmacophore and molecular docking based screening

The screening workflow described in our previous study was used^17^ to screening potential HDACis in the ZINC database^19^. As a result, 25 hit chemical candidates with different structures were screening out as final hit lead chemical candidates for further experimental validation.

### Inhibitory enzymatic activity evaluation

**Figure 1:**
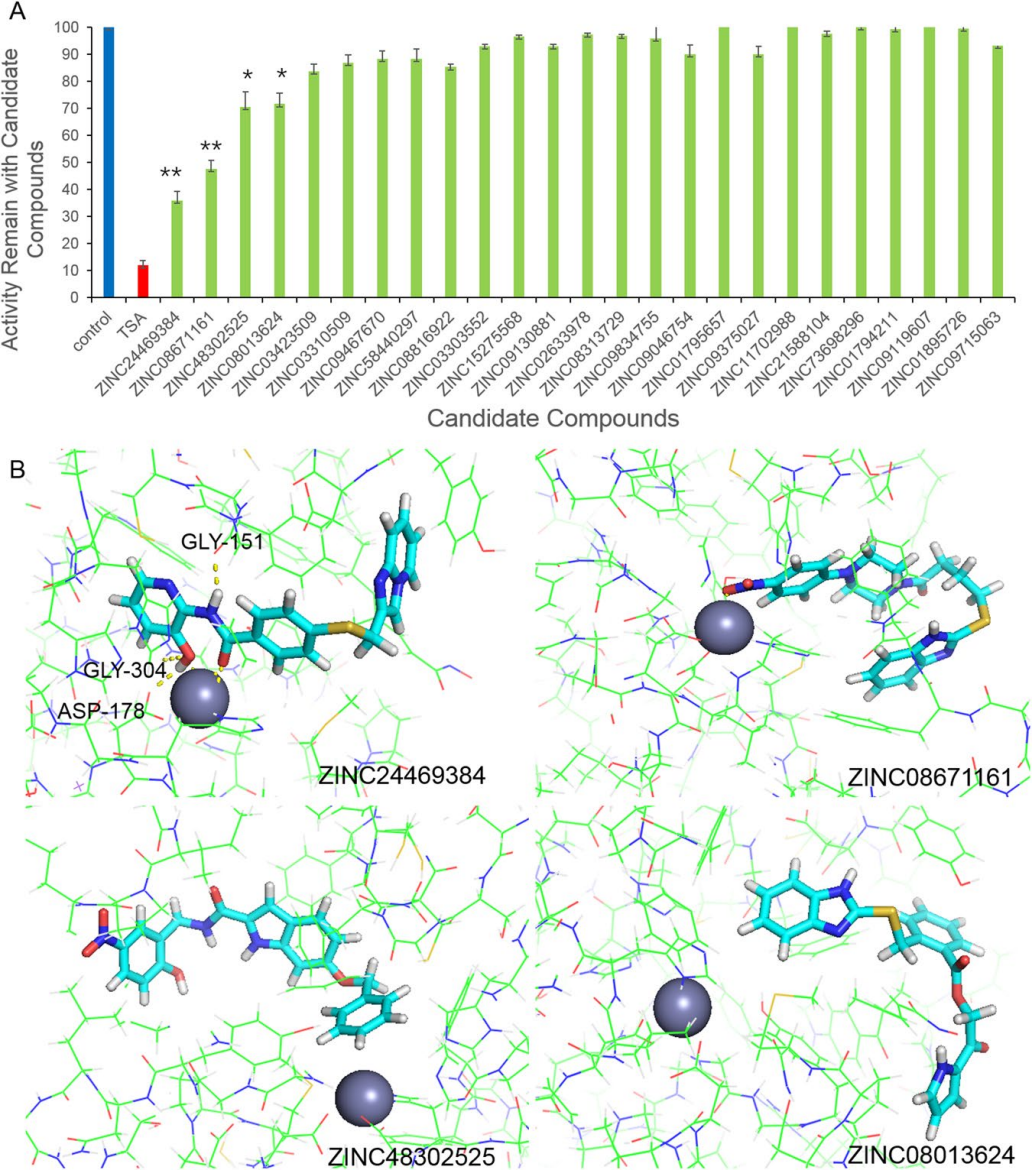
Inhibitory effect of the 25 hit lead candidates on HDAC and molecular docking results of the four active lead candidates. (**A**) The HDAC activities ratio of the hit lead candidates. The positive control was represent by blue, the inhibitor control was represent by red, the hit compounds was represent by green. The results are the mean ± S.D. of at least three independent experiments. Two-tailed student’s t-test was performed between HDACi vs. control, *P < 0.05, **P < 0.01. (**B**) Docking analysis of ZINC24469384, ZINC08671161, ZINC48302525 and ZINC080113624 to HDAC and a yellow dotted line shows the hydrogen bond.

We use a fluorometric HDAC activity kit to analysis the inhibition effect of the 25 lead compounds to HDACs. The results show that four compounds, namely ZINC24469384, ZINC08671161, ZINC48302525 and ZINC08013624 have inhibition effect to HDACs. After the compounds treated, the activity of enzymes were 36%, 47.6%, 70.6% and 83.6%, TSA was used as a control, the activity of enzyme was 11.5%. The other 21 lead chemical candidates does not show significant inhibitory effects in this experiment (Fig. 1A).

Among the four active lead compounds, only ZINC24469384 belongs to benzamide and the other three compounds do not belong to four main types of HDAC inhibitors^1^. The functional groups of ZINC24469384 were: hydroxypyridin, imidazo pyridine and benzamide, respectively; ZINC08671161 has three functional groups: benzo imidazol, nitrophenyl and piperazin; the functional groups of ZINC48302525 were: benzyloxy, nitrobenzyl and carboxamide, respectively; the functional groups of ZINC09715944 were: pyrrol, benzo imidazol and benzoate. The docking results of the four chemical candidates shown that the hydroxyl of benzamide in ZINC24469384 formed hydrogen bond with amino acid residues GLY-151, GLY-304 and ASP-178. For ZINC08671161, zinc ions can formed chelate with its nitrobenzene functional group. ZINC48302525 and ZINC09715944 can form non-polar interaction or VDW interaction with protein (Fig. 1B) and the four lead candidates were used for analyzing the anti-cancer effect.

### The mechanisms of anti-cancer effect of HDACi candidates

**Figure 2:**
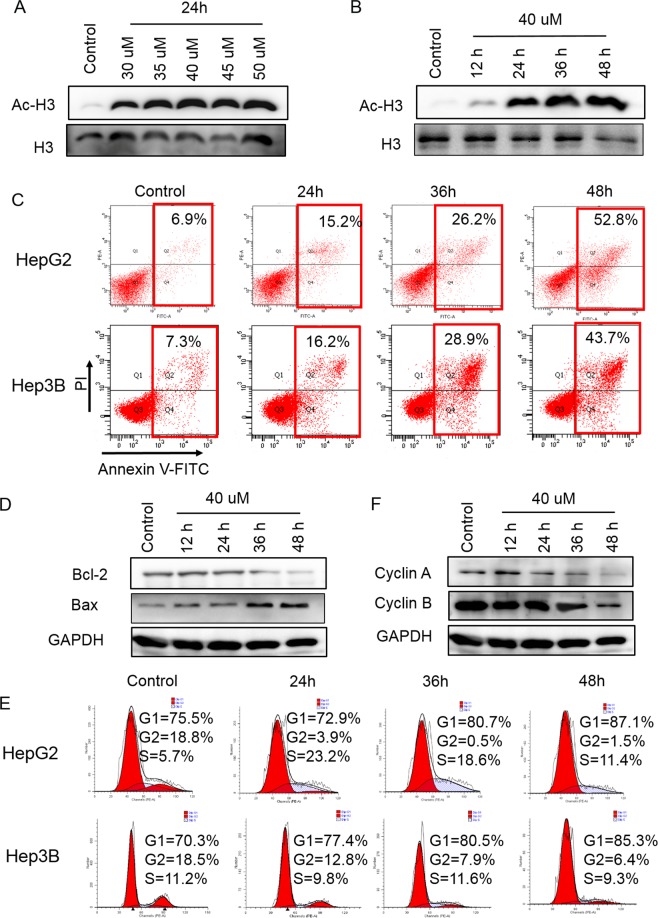
ZINC24469384 can induces apoptosis and cell cycle arrest in HepG2 cells. (**A**,**B**) Assessment of the dose dependent and time dependent effect of ZINC24469384 on acetylation histone H3 in HepG2 cells after treatment. H3 was used as an internal control. (**C**,**E**) HepG2 and Hep3B cells were treated with 40 μM control (DMSO) or ZINC24469384 for 24 h, 36 h and 48 h. Then the cells were stained with Annexin V^+^/PI^+^ or PI and using flow cytometer to analysis the percentage of apoptosis cells and cell cycle phase distribution. (**D**,**F**) HepG2 cells were treated with μM DMSO for 48 h or equivoluminal ZINC24469384 for 12 h, 24 h, 36 h and 48 h, respectively. Cells were collected and analysis the expression of apoptosis marker (Bax and Bcl-2) and cell cycle arrest markers (CyclinA and CyclinB) using Western Blot. GAPDH was used as an internal control.

HDACis specifically induce cell cycle arrest of cancer cell, initiate apoptosis and differentiation^20^. So we use MTT assay to test the anti-cancer effects of the four candidates. Five cancer cell lines and a normal cell line (HepG2, Hep3B, A549, A2780, SGC7901 and L02) cells were used and treated with ZINC24469384, ZINC08671161, ZINC48302525 and ZINC08013624 for 48 h, then the IC50 value of the four candidates against six cell lines were listed in Table 1. ZINC24469384 showed stronger inhibitory effects than other three candidates, the IC50 values of ZINC24469384 on HepG2, Hep3B, A549, A2780, SGC-7901 and L02 cells were 39.6 ± 5.74, 49.31 ± 4.58, 53.41 ± 1.51, 56.56 ± 4.66, 69.4 ± 4.15, and 79.47 ± 3.01 μM, respectively. The IC50 value of normal cell was two times higher than HepG2 cells IC50 value and almost two times higher than Hep3B cells IC50 value. The results show that the ZINC24469384 has anti-tumor effect against all five tumor cell lines, especially in liver cancer. ZINC08671161 and ZINC48302525 have anti-tumor effect against HepG2, Hep3B, A549, HeLa and A2780 cell lines but can they also have stronger toxicity towards the normal cell line L02. ZINC08013624 has no anti-cancer effects against five cancer cell lines. Western Blot showed that ZINC24469384 can cause, hyperacetylation of histone in chromatin in a time-dependent and dose-dependent way in HepG2 cells (Fig. 2A,B), the band intensities were measured compared to H3 control (Supplementary Fig. S1A,B). The above results indicated that ZINC24469384 has anti-tumor effect against cancer cells, especially in liver cancer cell lines and it can also increase the acetylation level in cancer via a time/dose dependent way.

**Table 1:** The IC50 values of ZINC24469384, ZINC08671161, ZINC48302525 and ZINC080113624 against different cancer and normal cell lines.

Chemicals	HepG2	IC50(μM)				
		Hep3B	L02	A549	A2780	SGC7901
ZINC24469384	39 ± 5	49 ± 4	79 ± 3	53 ± 1	56 ± 4	69 ± 4
ZINC08671161	124 ± 17	151 ± 20	70 ± 3	154 ± 31	103 ± 5	>300
ZINC48302525	115 ± 5	232 ± 26	152 ± 22	258 ± 22	128.4 ± 8	>300
ZINC080113624		>300

Then, we detected the effect of ZINC24469384 on induce apoptosis by flow cytometry in liver cancer cells. Base on the IC50 value of ZINC24469384 on HepG2 and Hep3B cells, 40 μM was used as the apposite working concentration for analysis. The two liver cancer cell lines were treated with ZINC24469384 for 0 h, 24 h, 36 h and 48 h, respectively. The results showed that, ZINC24469384 can significantly induced the apoptosis in the two liver cancer cells. The percentage of apoptosis cells in HepG2 after treated with different times intervals were 6.9%, 15.2%, 26.2% and 52.8%, respectively. The apoptosis rate of Hep3B cells treated with different times intervals were 7.3%, 16.2%, 28.9% and 43.7%, respectively (Fig. 2C). These results illustrated that apoptosis induced by ZINC24469384 was time dependent.

To know whether apoptosis induced by ZINC24469384 was modulated by Bcl-2 family members, further Western Blot was performed and the results showed that ZINC24469384 downregulated the expression of Bcl-2 and upregulated the expression of Bax in HepG2 cells (Fig. 2D), and they increased via time-dependent way (Supplementary Fig. S1C,D). Moreover, ZINC24469384 can decreased ATP levels time dependently in HepG2 cells and also induced changes in morphology such as chromatin condensation and apoptotic bodies (Supplementary Fig. S1E,F). All these data illustrated that ZINC24469384 can induce apoptosis of HepG2 cell in time-dependent manner and partly by intrinsic (mitochondrial) pathway.

HDACi can not only induce cell apoptosis, it can also inhibit cell proliferation^3^. Furthermore we also analysis the distribution of cell cycle phases after treated with ZINC24469384 by flow cytometric. Analysing the results, we could find that after exposure to ZINC24469384 for 0 h, 24 h, 36 h and 48 h, the G1/S phase significantly increased by 14.9%, 18.3% and 17.3% in HepG2 cells. And in Hep3B cells, the percentage of G1/S phase significantly increased by 5.7%, 10.6% and 12.1%, respectively (Fig. 2E). Since Cyclin A and Cyclin B play an important role in regulating G1 and S cell cycle phase^21^, further Western Blot was performed to analysis the expression of Cyclin A and Cyclin B. As shown in data, the expression of Cyclin A and Cyclin B decreased after ZINC24469384 treated (Fig. 2F), in addition, they all decreased via time-dependent way (Supplementary Fig. S1G,H). Taken together, these results indicated that ZINC24469384 can promote apoptosis of cancer cells and selectively inhibit proliferation.

### After ZINC24469384 treated the DEGs and DASGs were functionally coupled

**Figure 3:**
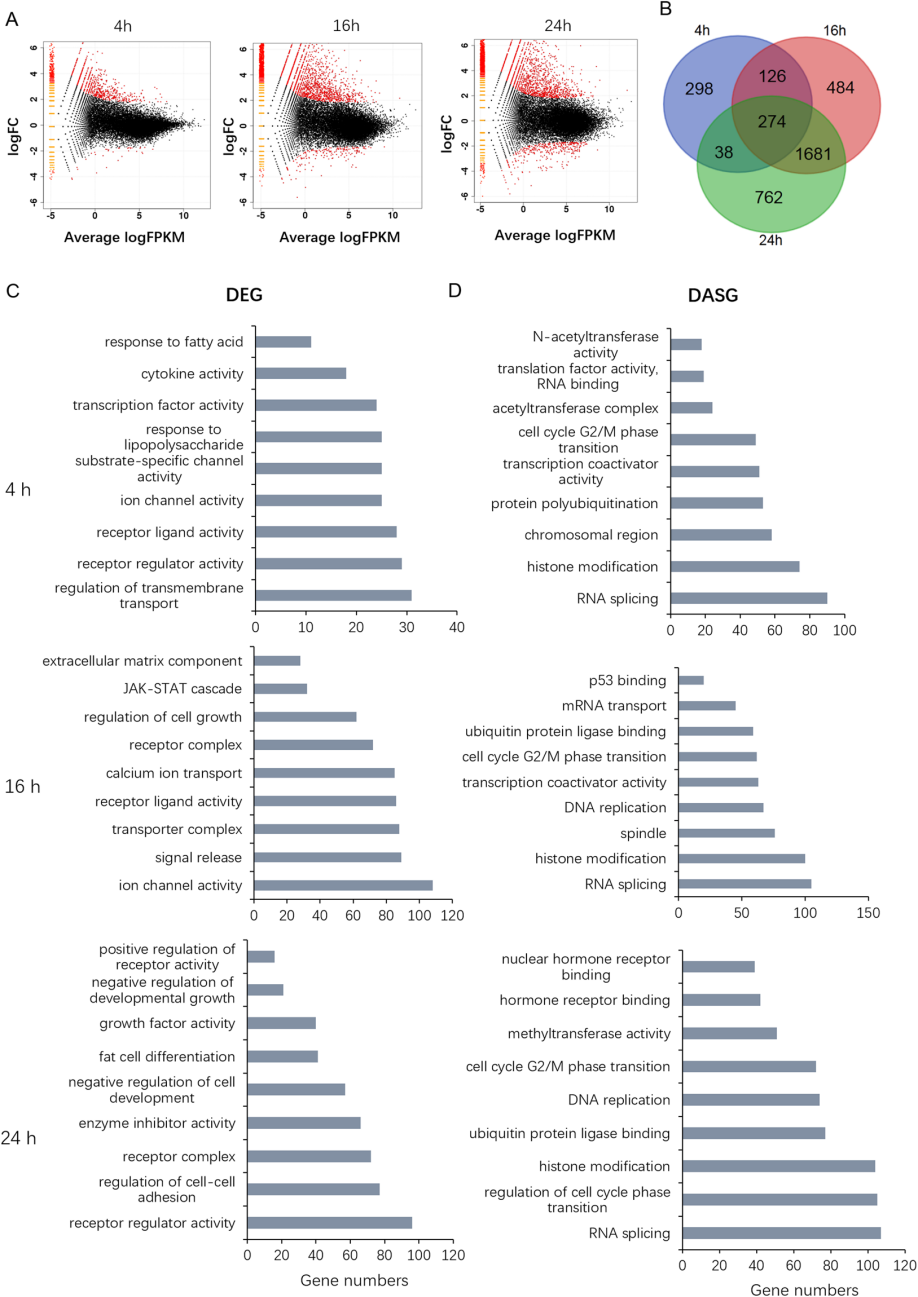
GO enrichment analysis of DEGs and DASGs after ZINC24469384 treated. (**A**) Volcano Plot of the DEGs after ZINC24469384 treated (4 h, 16 h and 24 h) vs. control treated (4 h, 16 h and 24 h). P < 0.01 and fold change ≥2 or fold change ≤1/2. (**B**) Venn diagram showing the DEGs (fold change ≥2 or fold change ≤1/2) after ZINC24469384 treated for 4 h, 16 h and 24 h. (**C**) Functional annotations of DEGs by ClusterProfiler after ZINC24469384 treated for 4 h, 16 h and 24 h. (**D**) Functional annotations of DASGs by ClusterProfiler after ZINC24469384 treated for 4 h, 16 h and 24 h.

ZINC24469384 can cause anti-cancer effects in liver cancer cells, while detailed molecular mechanism of ZINC24469384 could suppress the viability of liver cancer cell need to be further elucidated. We used RNA-seq to globally understand of the changes of HepG2 cells after incubation with DMSO or ZINC24469384 for 0 h, 4 h, 16 h and 24 h, respectively. The number of differentially expressed genes (DEGs) were 737 genes, 2565 genes and 2755genes respectively (fold change >2, P < 0.05), the DEGs were enriched in upregulated genes (Fig. 3A). The overlap between genes altered across the three treatment times and 274 genes were altered in all three time points (Fig. 3B). Then we analysis the functional classification of the DEGs after 4 h, 16 h and 24 h treated, through classification into gene ontology (GO) categories. The genes response to 4 h treatment were associated with response to fatty acid, receptor ligand activity, receptor regulator activity and transcription factor activity; after 16 h treatment, the genes were involved in signal release, JAK/STAT cascade and receptor ligand activity; then after 24 h treatment, the DEGs were involved in enzyme inhibitor activity, positive regulation of receptor activity, negative regulation of growth and receptor regulator activity were also identified (Fig. 3C). The alterations in GO categories indicated the anti-cancer effect of ZINC24469384 may associated with response to fatty acid, JAK/STAT cascade, regulation of receptor ligand activity and negative regulation of growth.

We all know that many anti-tumor drugs can inhibit cancer cell growth via altering gene alternative splicing^22–24^. Lots of studies indicated that gene transcription and alternative splicing might be functionally cross-talked, and contribute to the pharmacological actions of drug together^24,25^. So alternative splicing analysis were used in our analysis. The data indicated that after ZINC24469384 treated, the number of Differentially Alternative Spliced Genes (DASGs) were 1427 genes, 1907genes and 2036 genes respectively (Incleveldifference >5%, P < 0.05), the DASGs were enriched in Skipped Exon genes (Supplementary Table 1). Then we also analyzed the functional classification of DASGs, through classification into gene ontology (GO) categories. The genes response to 4 h treatment were associated with response to RNA splicing, histone modification and cell cycle G2/M phase arrest; After 16 h treatment, the genes were involved in RNA splicing, histone modification and cell cycle G2/M phase arrest, P53 binding; then after 24 h treatment, the genes were involved in RNA splicing, regulation of cell cycle arrest and histone modification were also identified (Fig. 3D). Above all, the results showed that the anticancer action of ZINC24469384 might be DEGs and DASGs functionally coupled.

### ZINC24469384 affects essential molecular pathways in HepG2 cells

**Figure 4:**
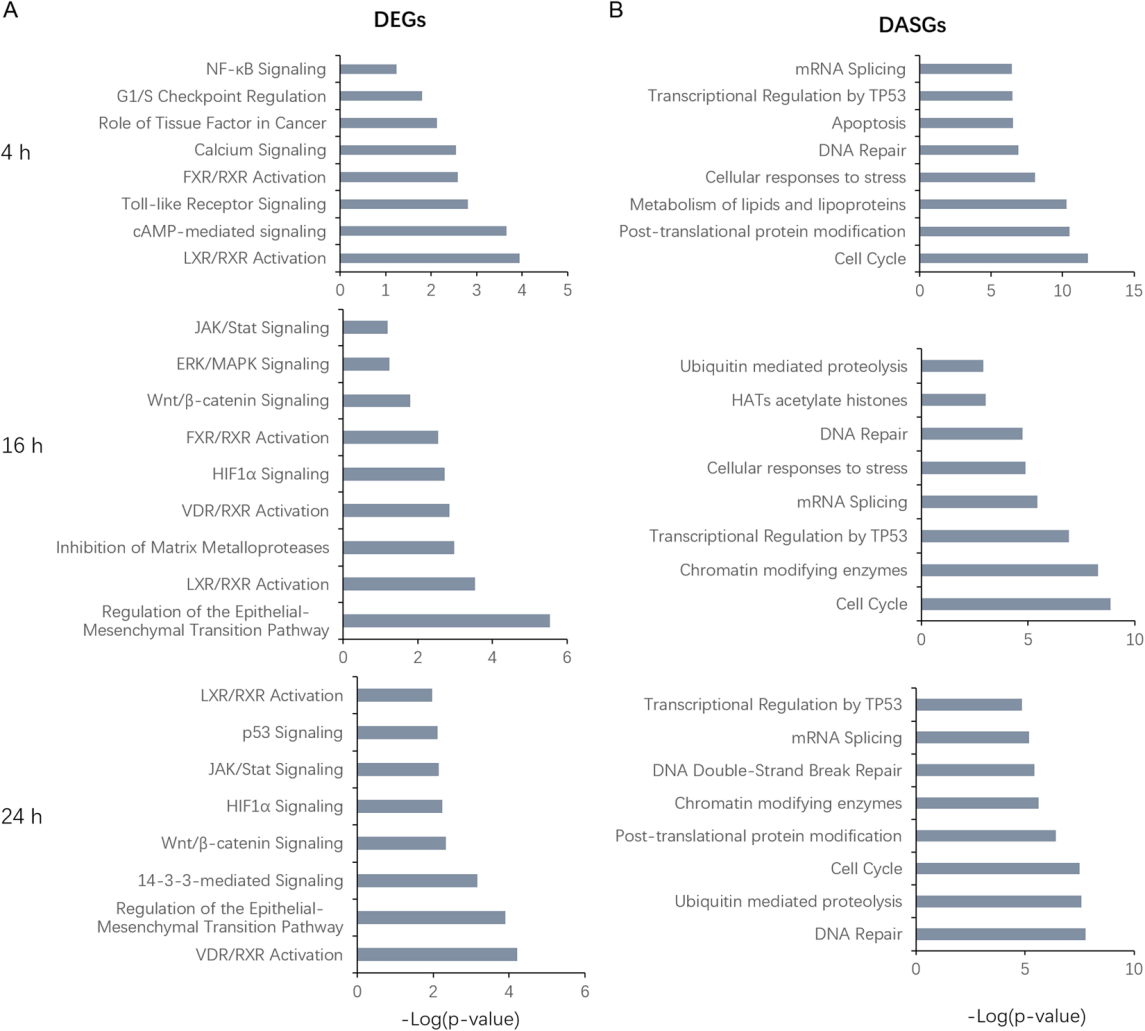
Pathway enrichment of DEGs and DASGs after ZINC24469384 treated in HepG2 cells. (**A**) IPA canonical pathways of DEGs after ZINC24469384 treated for 4 h, 16 h and 24 h. (**B**) Pathway analysis of DASGs after ZINC24469384 treated for 4 h, 16 h and 24 h, Incleveldifference >5%, P < 0.05.

To know the pathways affected by treated with ZINC24469384. The DEGs and DASGs generated from each individual treatment were used for pathway enrichment analysis, the results showed that, after exposure to ZINC24469384 for 4 h, DEGs were associated with FXR/RXR activation, Toll-like receptor signaling and G1/S checkpoint regulation; after continued exposure to ZINC24469384 for 16 h, DEGs were associated with Glutamate receptor signaling, LXR/RXR activation, FXR/RXR activation and JAK/STAT signaling; then after continued exposure to ZINC24469384 for 24 h DEGs were associated with regulation of the EMT pathway, 14-3-3-mediated signaling, JAK/STAT signaling, P53 signaling and LXR/RXR activation (Fig. 4A). Meanwhile, the pathway analysis of DASGs showed that, after exposure to ZINC24469384 for 4 h, DASGs were associated with mRNA splicing, cell cycle, metabolism of lipids and lipoproteins and transcription regulation by TP53; after continued exposure to ZINC24469384 for 16 h, DASGs were associated with cell cycle, transcriptional regulation by TP53, mRNA splicing, DNA repair and HATs acetylate histones; then after continued exposure to ZINC24469384 for 24 h, DASGs were associated with regulation of DNA repair, cell cycle, mRNA splicing and transcriptional regulation by TP53 (Fig. 4B).

**Figure 5:**
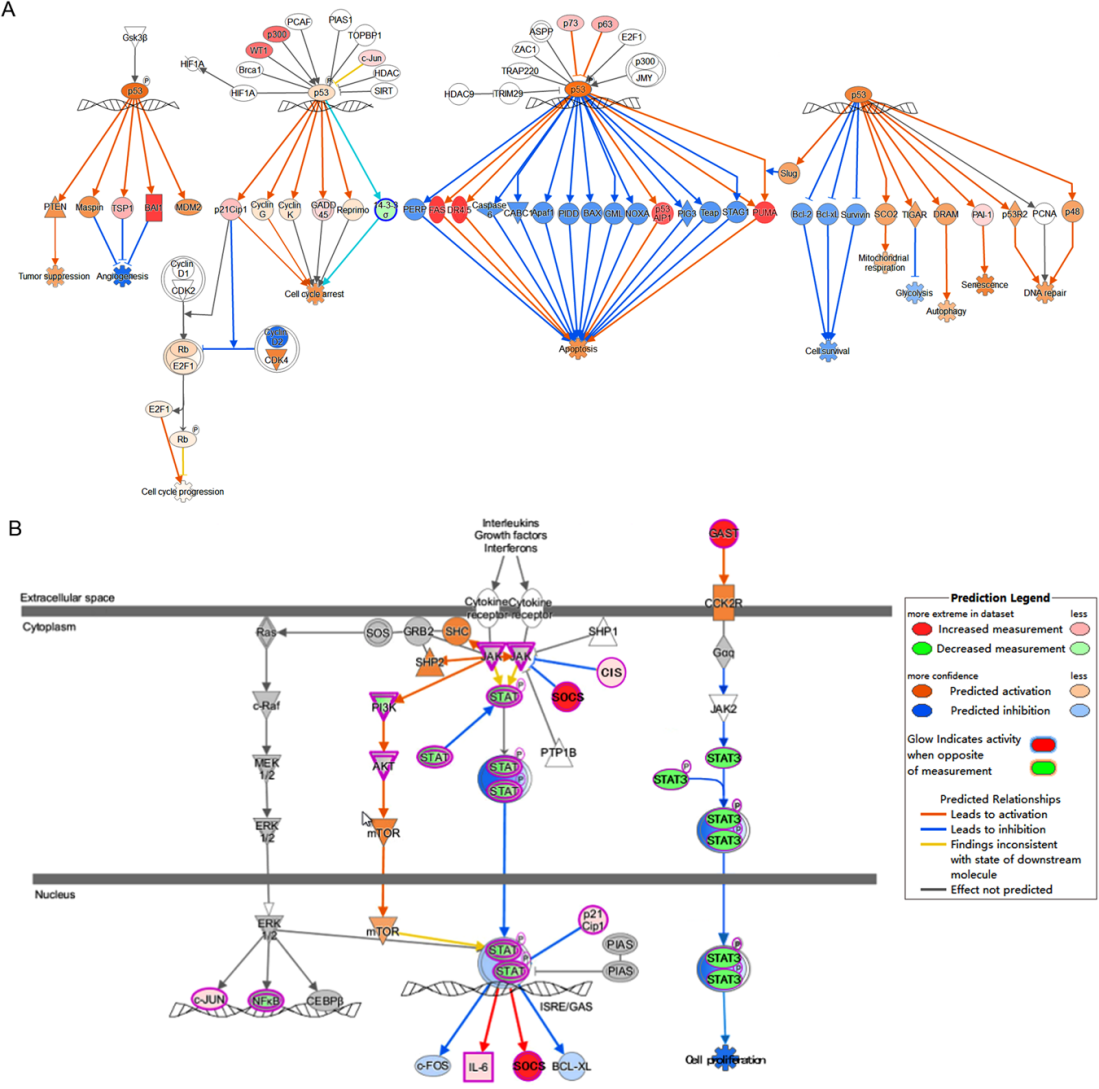
ZINC24469384 regulates genes involved in P53 and STAT signaling pathways. (**A**) The molecular activate prediction of P53 pathway using genes altered by three time points. (**B**) The molecular activate prediction of STAT signaling pathways using genes altered by three time points. Different shapes represent different classes of protein, upregulation was represented by red, downregulation was represented by green, direct interaction was represented by solid lines and indirect interactions was represented by dashed lines. The pathways were generated via IPA (QIAGEN Inc., https://www.qiagenbioinformatics.com/products/ingenuity-pathway-analysis/)^51^.

The potential mechanisms of the anti-tumor effects of ZINC24469384 were proposed based on the pathway enrichment analysis. Six genes (GADD45α, CDKN1A, BAI1, CITED1, BMF and BIK) were up-regulated and four genes (CCNB1, CCNA2, CDK2 and CDC25A) were down-regulated in p53 signaling pathway and the genes can be regulated by p53^26^, as is known, BAI1 play a key role in anti-angiogenesis and p53 is an important checkpoint protein in regulating the cell cycle transition^27^ (Fig. 5A); Active the STAT signaling negative regulators (SOCS1, SOCS2) are another important way for anti-proliferation (Fig. 5B). Overexpression of STAT3 plays a key role in tumor proliferation, lots of studies indicated that inhibit the activation of STAT3 is beneficial for cancer treatment, so the expression of SOCS1, SOCS2 may inhibit tumor cell survival^28^. The categories also be driven by up-regulated FXR/RXR activation associated genes (NR1H4, SULT2A1, MDR3, HNF4α). In previous study, NR1H4 plays a key role in lipid and glucose metabolism, while, recently study indicated that NR1H4 it also participate in protecting against liver cancer^29^. NR1H4 is also associate with both FXR/RXR and LXR/RXR activation^30^, highly expressed of NR1H4 in liver can protecting against liver cancer^29^. Activation of it can inhibit cell growth, arrest cell at G1 phase^31^ and upregulate the expression of HNF4A, which mediates the regeneration of liver^32^. It can also upregulate the expression of STAT signaling negative regulator SOCS3^31,33^, this indicated that NR1H4 might be the upregulator of SOCS3. In our data SOCS family numbers SOCS1 and SOCS2 were upregulated after ZINC24469384 treated which means SOCS1 and SOCS2 may also be new target genes of NR1H4. This suggested that NR1H4-SOCS signal was likely to play a substantial role in anti-cancer effect after ZINC24469384 treated.

**Figure 6:**
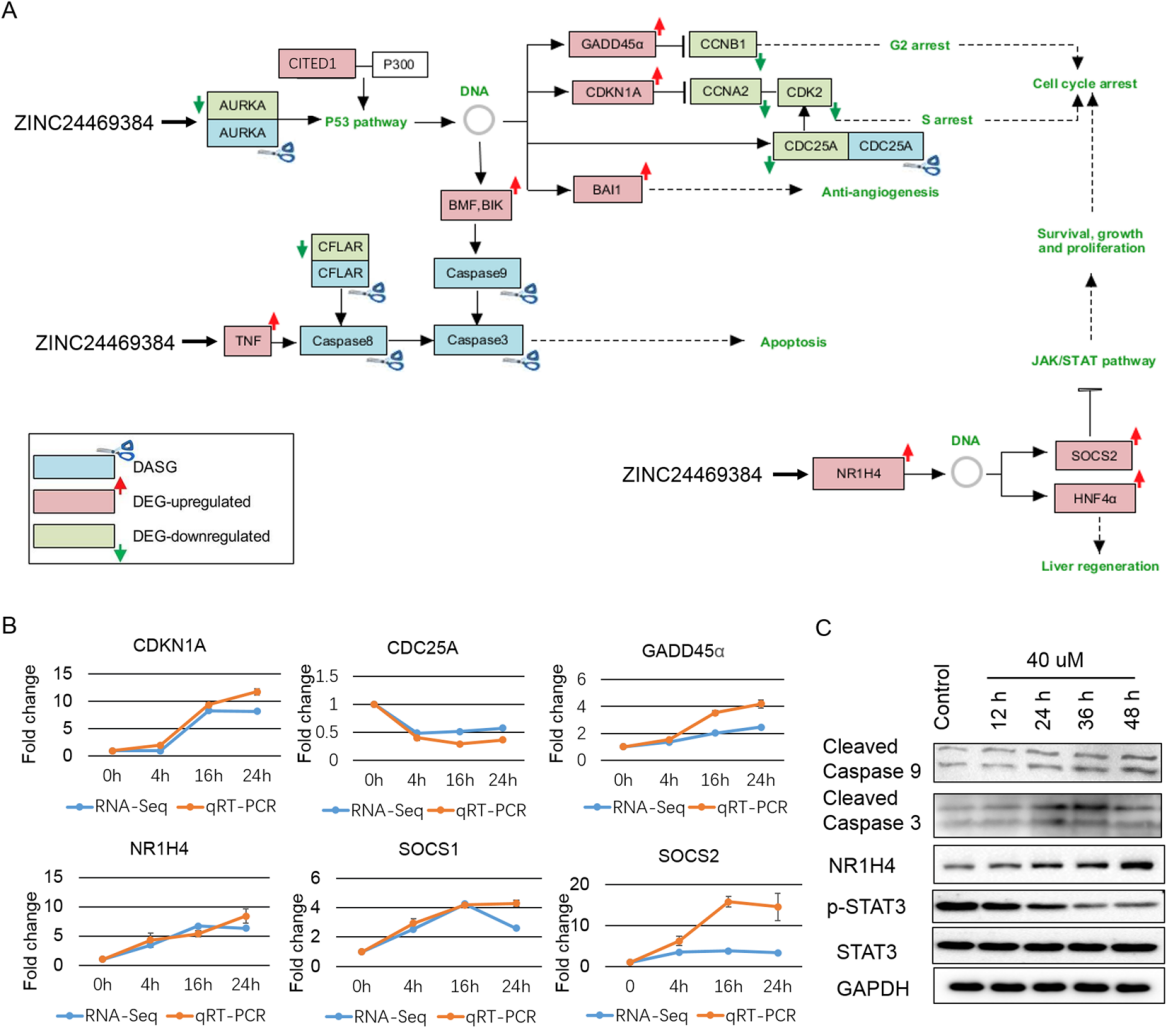
The DEGs and DASGs induced by ZINC24469384 were functionally cross-talked. (**A**) A merged network include DEGs and DASGs were build based on p53 signaling pathway, JAK/STAT pathway and apoptosis. (**B**) The mRNA expression of CDKN1A, CDC25A, NR1H4, GADD45α, SOCS1 and SOCS2 in HepG2 cells after ZINC24469384 treated for 4 h, 16 h and 24 h in HepG2 cells. The values calculated three independent experiments and show as mean ± SD. (**C**) The expression of Cleaved Caspase 9, Cleaved Caspase 3, NR1H4 and p-STAT3 in HepG2 cells using Western Blot after ZINC24469384 treated for 4 h, 16 h and 24 h in HepG2 cells. GAPDH and STAT3 were used as loading control. The values calculated three independent experiments and show as mean ± SD.

According to the pathway analysis on those DASGs, TP53 signal pathway and cell cycle related pathways were affected after ZINC24469384 treated, five genes (AURKA, CDC25A, Caspase8, Caspase9 and Caspase3) were alternatively spliced, and the genes were at the upstream of p53 pathway, cell cycle arrest and apoptosis. Serine/threonine kinase (AURKA) can phosphorylate p53 and regulate the expression of multiple mitosis-associated proteins^34,35^; CDC25A is a cell cycle checkpoint protein, play a key role in cell cycle transition. This indicated that DASGs induced by ZINC24469384 may associated with the proliferation of liver cancer. These data shown that DEGs and DASGs might be functionally connected. A merged pathway network involved DEGs and DASGs was generated based on apoptosis, p53 signaling pathway and JAK/STAT pathway (Fig. 6A), the potential functional cross-talk between DEGs and DASGs were indicated. To validate the RNA-seq results and our pathway hypothesis, real-time PCR was used to confirm the expression of CDC25A, CDKN1A, GADD45α, NR1H4, SOCS1 and SOCS2 (Fig. 6B); we also use Western Blot to confirm the expression level of NR1H4 and P53 were significantly increased, phosphorylated-STAT3 was significantly decreased, in addition, Caspase-3 and Caspase-9, two mitochondria-related apoptosis proteins were also activated (Fig. 6C). The Western Blot band intensities were measured compared to H3 or STAT3 as control (Supplementary Fig. S2A,B). So these results strongly indicated that the DEGs and DASGs induced by ZINC24469384 might be work together and functionally connected.

### NR1H4 is involved in the anti-cancer effect of ZINC24469384

In our study, ZINC24469384 can selectivity induced apoptosis in cancer cells, especially in liver cancer, to know the molecular mechanism why it can selectivity against liver cancer, we analysis the expression of 382 genes specially expressed in liver^36^. Among the genes, 48 genes were differently expressed after ZINC24469384 treated, five genes (NR1H4, HNF4A, IL1B, NR5A2 and AREG) were upregulated in all three time points (Supplementary Fig. S3). Three of the five genes (NR1H4, HNF4A and NR5A2) were participate in FXR/RXR activation pathway and the fold change of NR1H4 was more significant than other genes. So NR1H4 might play critical role in ZINC24469384 induced selectivity against liver cancer.

**Figure 7:**
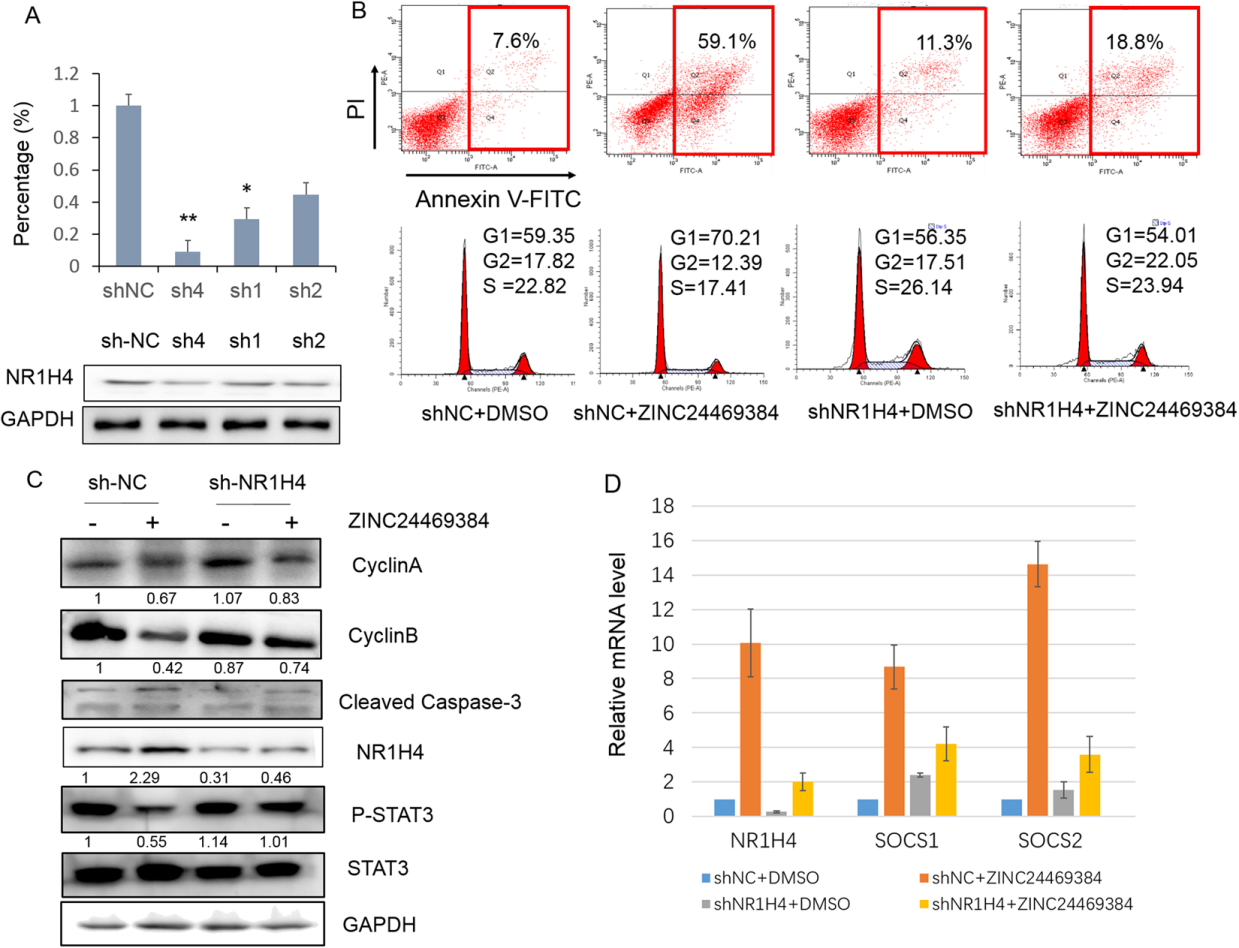
ZINC24469384 influences anti-cancer effects in HepG2 cells may partly through NR1H4. (**A**) HepG2 cells were transfected with the shRNA targeting NR1H4 for 48 h and selected with puromycin (1 μg/ml) for four days, the expression of NR1H4 was measured by RT-qPCR and Western Blot. (**B**) Transformed HepG2 cells were incubate with 40 μM control (DMSO) or ZINC24469384 for 48 h, then the cells stained with Annexin V^+^/PI^+^ or PI and the percentage of apoptosis cells or cell cycle phase were measured by a flow cytometer. (**C**) Transformed HepG2 cells were incubate with 40 μM control (DMSO) or ZINC24469384 for 48 h, then using Western blot to analysis the expression of apoptosis, cell cycle arrest associated proteins and phosphorylation of STAT3. GAPDH and STAT3 were used as loading control. The values calculated three independent experiments and show as mean ± SD. (**D**) Transformed HepG2 cells were incubated with 40 μM ZINC24469384 or control (DMSO) for 48 h, and the expression of NRIH4, SOCS1 and SOCS2 were examined by RT-qPCR.

To investigate whether NR1H4 is involved in apoptosis induced by ZINC24469384, we constructed three RNAi plasmids targeting NR1H4 gene and shRNA-4 was more efficient than other vectors (Fig. 7A), we also measured the Western Blot band intensities (Supplementary Fig. S2C). Thus, NR1H4 shRNA-4 was used in the following research. We used NC-shRNA or NR1H4 shRNA-4 HepG2 stably transfected HepG2 cells treated with ZINC24469384 for 48 h then using flow cytometric to analysis. The data showed that knockdown of NR1H4 induced significant anti-apoptosis effect after ZINC24469384 treatment compared with DMSO (59.1% ± 0.27% vs. 18.8% ± 0.38 in NR1H4-shRNA stably transfected cells) (Fig. 7B).

We also used NC-shRNA or NR1H4 shRNA-4 stably transfected HepG2 cells, to detect the distribution of cell cycle phase by flow cytometric analysis. NC-shRNA and NR1H4-shRNA stably transfected cells were incubated with ZINC24469384 or control (DMSO) for 48 h then detected by flow cytometry. Down-regulation of NR1H4 prominently inhibited ZINC24469384 induced anti-proliferation effect of HepG2 cells (Fig. 7B). Moreover, decreased expression of NR1H4 increased cell population in G1 phase and reduced the percentage of cells in S phase after ZINC24469384 treatment. We further used western blot to measure the apoptosis and cell cycle associated genes expression in NC-shRNA and NR1H4-shRNA stably transfected cells after treatment with DMSO or ZINC2446938. As expected, cleaved-caspase-3 was upregulated after ZINC24469384 treated in NC-shRNA stably transfected HepG2 cells but not in NR1H4 shRNA-4 stably transfected cells; while the expression of cyclinA and cyclinB reduced by ZINC24469384 were significantly upregulated by RNAi the NR1H4 gene (Fig. 7C). These observations convincingly suggest that NR1H4 may partly contribute to ZINC24469384-mediated apoptosis and cell cycle arrest in liver cancer cells.

Furthermore there was a study suggesting that activation of NR1H4 can induce the expression of SOCS3 the negative regulator to JAK/STAT pathway^33^, it regulates many growth signaling which use STAT superfamily numbers like STAT3 to promote cell growth and apoptosis^37^. We next assessed whether ZINC24469384 induced upregulation of NR1H4 can inhibit phosphorylation of STAT3. Treatment with ZINC24469384 in NC-shRNA stably transfected HepG2 cells caused an apparent diminution effect on phosphorylation of STAT3, however, the phosphorylation of STAT3 was dramatically stimulated when the NR1H4 gene was downregulated, which suggests a failure in response to ZINC24469384 (Fig. 7C). In a previous study, NR1H4 can inhibit STAT3 activation by upregulating SOCS3^33^, we also assessed the gene expression of the other SOCS family numbers in transformed cells. Knockdown of NR1H4 can also attenuate the expression level of SOCS1 and SOCS2 after ZINC24469384 treatment, and the decrease of SOCS2 is sharper than SOCS1 (Fig. 7D), These data indicated that SOCS2 may play more important role in NR1H4 induced anti-tumor effects. The above results suggest that ZINC24469384 possesses an anti-proliferation effect via a possible mechanism of inducing expression of NR1H4 then up-regulating the downstream genes SOCS2 and inhibiting STAT3 activation *in vivo*.

### NR1H4 and SOCS2 is associated with human hepatic celluler cancer

We also data mining using public TCGA data sets to examine the expression of NR1H4 and SOCS2 in Liver Hepatocellular Carcinoma (LIHC) and Cholangiocarcinoma. Cholangiocarcinoma (CHOL, the second most common hepatic malignancy after HCC)^38^. Statistical analysis with ANOVA multiple comparisons test showed that the expression of NR1H4 and SOCS2 in tumor were lower than normal tissues in two cancer types. Interestingly, we noticed that the expression of NR1H4 exhibited a more decrease in phase II, but SOCS2 showed significantly lower expression in both of the phase (Supplementary Fig. S4). All together, these results shown that downregulation of NR1H4 and SOCS2 might partly play a key role in promote liver cancer and they maybe used as potential new biomarkers for liver cancer diagnosis.

## Discussion

HDAC has become an important therapeutic target and inhibiting HDACs has shown promising results in cancer therapy. Although some HDACis has been approved and used for cancer therapy and there also have lots of HDACis tested in clinical trials, dose-limiting toxicities including thrombocytopaenia, nausea and fatigue were additionally observed^7^, so novel HDACis with low side effects are still urgently needed. In this study, we employed a hierarchical virtual screening protocol described in our last paper^17^ to find compounds that inhibits HDAC activity, 25 lead compounds, which were not analyzed for HDAC inhibitory. Then four lead compounds were screening out in HDAC inhibition test. Subsequently, we demonstrated the toxicity of the four lead chemical candidates to different cancer and normal cell lines. In agreement with the powerful inhibition of HDAC, ZINC24469384 demonstrated high anti-proliferative activity to HepG2 cell line but low toxicity to L02 cell line. Besides of *in vitro* cytotoxicity assay, it is better add animal assay to evaluate the safety and effectivity of candidate compound, in the future study, we will focus on validate the anti-cancer effectivity and metabolism of ZINC24469384 using HepG2 Xenograft model and mouse model^39^. Previous study indicated that the key mechanism of anti-proliferation effect of HDACi was induce cell cycle arrest^40^. In our study, flow cytometry data and Western blot analysis indicated that the G1/S population increased after 24 h treatment of ZINC24469384, meanwhile G1/S phase proteins expression including CyclinA and CyclinB are strongly decreased, previous studies have reported similar results^41^. Cell cycle arrest is known as the directional step before cell apoptosis while cell apoptosis is believed to be another important mechanism for HDACi to induce anti-tumor proliferation^42^. The flow cytometry, qRT-PCR and Western blot analysis indicated that ZINC24469384 increased the apoptosis rate of HepG2 cell, coupled with high expression of activated caspase 9 and Bax, while Bcl-2 was decreased. Our results showed that ZINC24469384 could arrest cell cycle at G1/S phase and significantly promote HepG2 cell apoptosis via mitochondrial-dependent apoptosis.

To search for specific pathway of ZINC24469384-induced HepG2 cell apoptosis, we performed time-coursed RNA-seq experiment. The data illustrated that DEGs and DASGs might be functionally connected. Bioinformatics analysis revealed that the FXR/RXR signaling pathway was activated, and the gene NR1H4 in FXR/RXR activation pathway is significantly upregulated. Previous study indicated that NR1H4 was a tumor suppress gene in different types of cancer and highly expressed in liver^29^, and its expression was downregulated or silenced in lots of Stage I-IV tumors^43^. Furthermore overexpression of NR1H4 contributes to protect p53 and HNF4α from degradation^44,45^, and could decrease tumor size in mouse xenograft models^46^. While inhibit of NR1H4 can promote liver cancer cells proliferate and migrate via inducing cancer suppressor gene SOCS3 and inhibiting STAT3 activation^31^, which means negative regulators such as SOCS family number SOCS1 and SOCS2 may also be new target genes of NR1H4^29^. In our study, we indicated that ZINC24469384 can upregulated the expression of NR1H4, SOCS2 genes and reduce the phosphorylation level of STAT3 while after knockdown of NR1H4 in HepG2 cells substantially attenuated the ZINC24469384-mediated phenotype. Our analysis based on TCGA database indicated that NR1H4 and SOCS2 were downregulated in Liver Hepatocellular Carcinoma and Cholangio Carcinoma, this suggest NR1H4 and SOCS2 may play a key role in tumorigenesis. Activation of NR1H4-SOCS2 pathway may participate in these tumor suppression.

These results show that we find a new structure of benzamine HDACi-ZINC24469384, it is a promising bioactive lead compound of HDAC inhibitor and has anti-tumor potential. ZINC24469384 displayed a novel molecular mechanism in HDACi-mediated antiproliferation effects. Even though more researches are still needed, these results illustrated that NR1H4 is a negative regulator in liver cell proliferation and provides a new insight of HDACi for treatment in cancer that targeting NR1H4-SOCS2 signaling. It may be the basis for novel clinical therapy and could prevent or inhibit cancer cell growth at early stages.

## Methods

### Pharmacophore modeling and molecular docking

We used SYBYL-X 2.0^47^ for screen a group of ligand optimal conformations that combined low strain energy, steric overlap, and pharmacophoric similarity that described in our last paper^17^, then the pharmacophore modeling was used for database screening. Then we used GOLD 5.2^48^ for docking screening. The related research methods have been described in our last paper^17^.

### Cell culture and Cell proliferation assay

HepG2, Hep3B, SGC7901, A549, MDA-MB-231 and L02 cell lines were maintained in DMEM (Corning, USA) with 10% FBS (TBD, China) and 200 U/ml gentamycin (Grbio, China), all six cell lines were incubated in 5% CO2 incubator at 37 °C. We use MTT assay to analysis the cell proliferation, then 490 nm was used as the wavelength to measure the optical densities (ODs) by immunosorbent assay reader. The expression of inhibition ratio is as following: (1-OD value of test group/OD value of control) × 100%^49^. The data were displayed by mean ± standard deviation (S.D.).

### *In vitro* HDAC enzymatic activity evaluation

We use HDACi screening kit (BioVision, K340, USA) to identify the lead compounds which have HDAC inhibitory activities. The related research methods have been described in the reference^17^. The enzymatic activities ratio is as following: (OD value of test HDACi/OD value of water) × 100%. The data was displayed by the means of mean ± standard deviation (S.D.).

### Cell apoptosis and cell cycle analysis

To quantify cell apoptosis and cell cycle, HepG2 and Hep3B cells were cultured, then incubated with 40 μM of ZINC24469384. After 0 h, 24 h, 36 h and 48 h incubation, cells were collected, then stained with the Annexin V^+^ and PI^+^ (Roche) for 15 min at room temperature or fixed in 75% alcohol at 4 °C contain 12 hours, then treated with RNAse and stained with PI (Roche) for 30 min at 37 °C. The percentage of cells were measured by a flow cytometer, the cells with Annexin V^+^ and PI^−^ were defined as early apoptotic cells; the cells with Annexin V^+^ and PI^+^ cells were defined as late apoptotic cells. Total apoptosis cells were the sum of all the early apoptotic cells and the late apoptotic cells. The value of different cell cycle phases were analysed with Modfit software (Verity Software House, Topsham).

### Measurement of Mitochondrial Activities

To measure the activity of mitochondrial we quantify the concentration of ATP, cells were cultured in DMEM, then treated with DMSO or ZINC24469384 for 0 h, 6 h, 12 h, 24 h and 36 h, respectively. The concentrate of ATP was measured by a ATP determination kit (Beyotime, China). And a protein qualification kit (Beyotime, China) was used to express the ATP level as nmol/mg protein.

### RNA sequencing and analysis DEGs/DASGs

mRNA was extracted after cells were incubated with control (DMSO) or ZINC24469384 for 0 h, 4 h, 16 h and 24 h, respectively. mRNA-seq assays were performed using Illumina HiSeq 2000 system by Novogene (Beijing, China). The FASTQC Software was used to exam the raw data. The reads (quality score >28) were mapped to human genome (hg19), according to the genome annotation, uniquely localized reads were used for the DEG analysis. EdgeR were used to analyze the DEGs (fold change ≥2 or fold change ≤1/2) and MATs was used for detecting differential alternative splicing genes (DASG) (p < 0.05, Inclevel difference >0.05). GO analysis was generated through R Package: ClusterProfiler^50^. Then pathway analyses were generated through Ingenuity Pathway Analysis (QIAGEN Inc., https://www.qiagenbioinformatics.com/products/ingenuity-pathway-analysis/)^51^.

### Quantitative real-time PCR

Real-time PCR (at 3 min for 94 °C, 35 cycles at 94 °C for 15 sec and at 60 °C for 50 sec) was performed using SYBR Green PCR MasterMix (Roche). Melting curve analysis was used to distinguish whether the PCR products were specific or non-specific. The housekeeper gene GAPDH was used as an internal control. The data using the 2 −ΔΔCT method that was determined as previously described to analysis the results^52^.

### SDS-Page and Western Blot

The extraction of cytosol and nucleus proteins was performed using protein extraction sample kit (Sigma). The proteins were separated on SDS polyacrylamide gels electrophoresis and transferred to polyvinylidene fluoride membranes. Then we use 5% nonfat milk to block the membranes for 30 min, incubate with primary antibodies for 4 h (Cell Signaling), incubate with secondary antibody (Abcam) for 30 min, after that detect the proteins using ELC kit (Beyotime). GAPDH and H3 were used as an internal control.

### RNA interference assay

We use lentivirus to express short hairpin RNA (shRNA) targeting NR1H4 and negative control: scrambled shRNA were constructed by Cyagen (Suzhou, China). The targeting senses of the shRNAs were: shRNA hNR1H4#1: 5′-GCCTCAGGAAATAACAAATAA-3′; shRNA hNR1H4#2: 5′-GCCTCTGGATACCACTATAAT-3′; shRNA hNR1H4#4: 5′-CCCAAGTTCAACCACAGATTT-3′. For long-term gene silencing, purified lentiviruses (shRNA hNR1H4#1, shRNA hNR1H4#2 and shRNA hNR1H4#4) were added to HepG2 cells using MOI value of 30. After incubation for 48 hours, HepG2 cells were selected with puromycin (1ug/ml) for four days fluorescence microscope and Western Blot were used to quantify the efficiency of lentivirus infection.

### Clinical analysis

We use cancer public data from The Cancer Genome Atlas (TCGA) (https://cancergenome.nih.gov/) compared with our RNA-seq expression data. Liver Hepatocellular Carcinoma data (LIHC, normal tissue 50, tumor tissue 374) and Cholangio Carcinoma (CHOL, normal tissue 9, tumor tissue 36) data were used for validation. We used FPKM data precalculated by TCGA and log transformed before analysis. R studio was used to analysis the normal data and different phases cancer data, p < 0.05 was considered as statistical significant.

### Statistical analyses

The experiments in our study were repeated at least three times and the results are presented as average ± standard. R studio was used for analyzing data and two-tailed student’s t-test was performed between control and each sample. For all the tests, 0.05 was accepted for statistical significance.

## Supplementary information

A novel benzamine lead compound of histone deacetylase inhibitor ZINC24469384 can suppresses HepG2 cells proliferation by upregulating NR1H4
