# Supplementary material for: A novel benzamine lead compound of histone deacetylase inhibitor ZINC24469384 can suppresses HepG2 cells proliferation by upregulating NR1H4
Source: Sci Rep. 2019 Feb 20;9:2350. doi: 10.1038/s41598-019-39487-6 (PMC6382829; doi:10.1038/s41598-019-39487-6)
Supplement: Supplementary file 1 — A novel benzamine lead compound of histone deacetylase inhibitor ZINC24469384 can suppresses HepG2 cells proliferation by upregulating NR1H4 [file 41598_2019_39487_MOESM1_ESM.docx]

**Supplementary Information**

**A novel benzamine lead compound of histone deacetylase inhibitor ZINC24469384 can suppresses HepG2 cells proliferation by upregulating NR1H4**

Qiuhang Song^1^, Mingyue Li^1^, Cong Fan^1^, Yucui Liu^1^, Lihua Zheng^2^, Yongli Bao^1^, Luguo Sun^1^, Chunlei Yu^2^, Zhenbo Song^1^, Ying Sun^1^, Guannan Wang^2^, Yanxin Huang^1*^ & Yuxin Li^2*^

*^1^ National Engineering Laboratory for Druggable Gene and Protein Screening, Northeast Normal University, Changchun 130024, China;^2^ Research Center of Agriculture and Medicine gene Engineering of Ministry of Education, Northeast Normal University, Changchun 130024, China.*

*To whom correspondence should be addressed. Email: [huangyx356@nenu.edu.cn](mailto:huangyx356@nenu.edu.cn), liyx486@nenu.edu.cn. Tel: 86-0431-8916-5922; Fax: 86-0431-8916-5917.

**Supplemental Table 1:**

**Supplemental Table 1.** Number of differentially alternative spliced genes after ZINC24469384 treated for 4h, 16h and 24h.

|  | 4h | 16h | 24h |
| --- | --- | --- | --- |
| Alternative3' SplicingSite | 225 | 285 | 301 |
| Alternative5' SplicingSite (A5SS) | 157 | 236 | 278 |
| Mutually exclusiveexons (MXE) | 81 | 134 | 127 |
| Retained Intron (RI) | 287 | 343 | 369 |
| Skipped Exon (SE) | 1183 | 1725 | 1868 |
| Union Genes | 1427 | 1907 | 2036 |

**Supplemental Figure S1:**

**
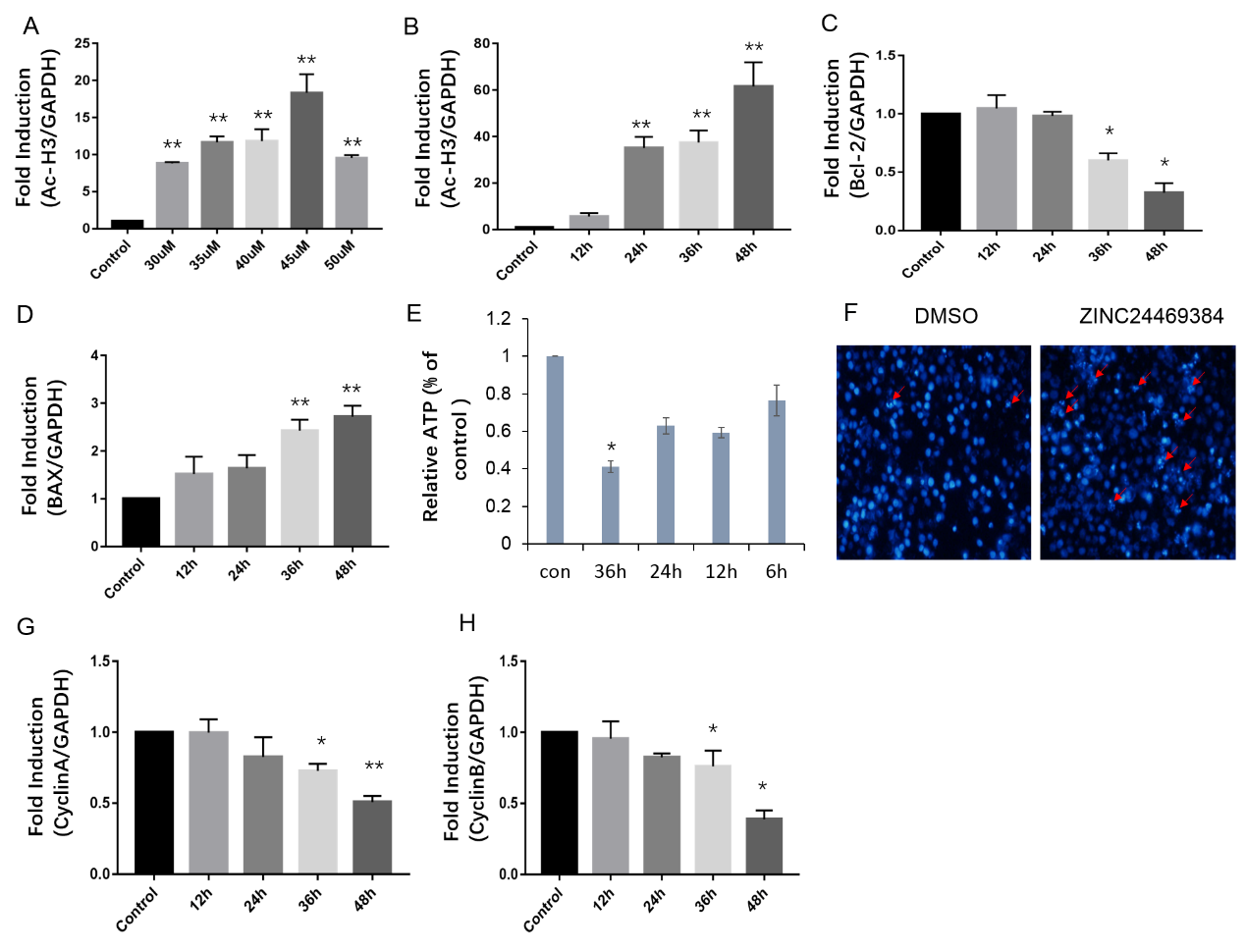
**

**Supplementary Figure S1. ZINC24469384 can induce apoptosis and cell cycle arrest in a time-dependent way.** **(A-E, G-H)** the quantitation of bands in figure 2 using Image J software. **(E)** The morphological changes of nuclear were visualized through DAPI staining. HepG2 cells treated with DMSO or ZINC24469384 (40 μM) for 48 h and stained with DAPI. Arrows indicate apoptotic nuclei. **(F)** HepG2 and BEL-7402 cells treated with ZINC24469384 (40 μM) for 0h, 6h, 12h, 24h and 36 h, the intracellular ATP content was performed using an ATP Assay Kit. Results were normalized to cellular protein concentration for each sample.

**Supplemental Figure S2:**

**
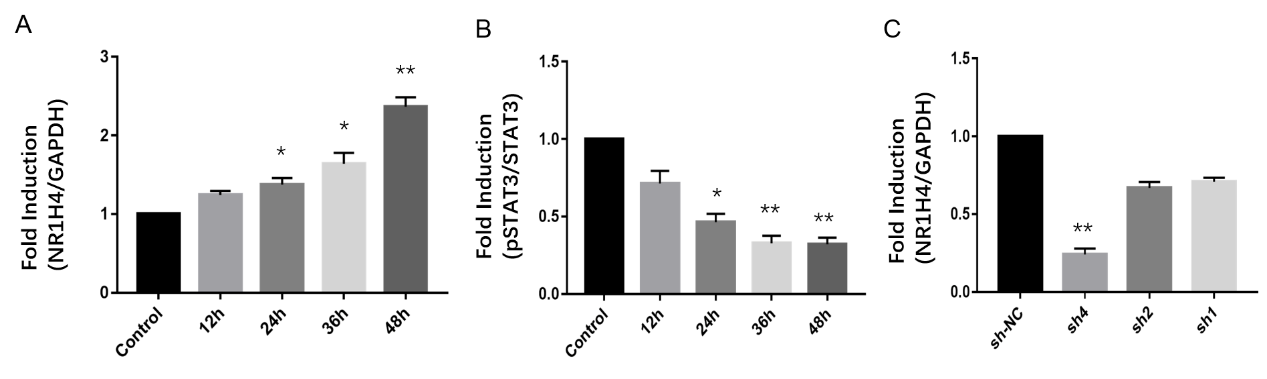
**

**Supplementary Figure S2. Quantitation of Western Blot bands. (A, B)** The quantitation of Western Blot bands in figure 6C using Image J software. **(C)** The quantitation of Western Blot bands in figure 7A using Image J software. The data represent three independent experiments.

**Supplemental Figure S3:**

**
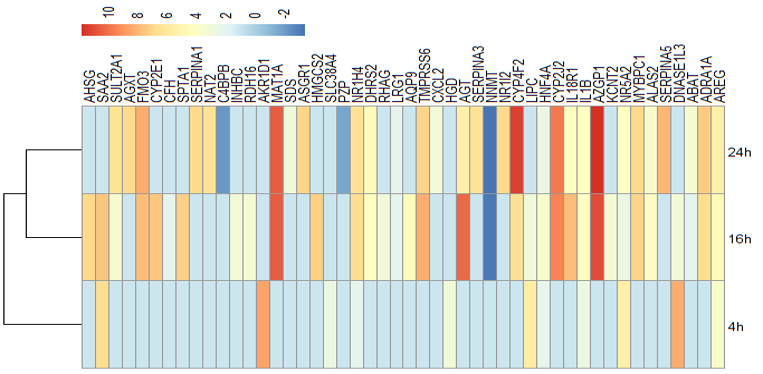
**

**Supplementary Figure S3. ZINC24469384 can induce genes specially expressed in live.** Heat map representing the liver specifically expressed genes were differently expressed after S24 treated.

**Supplemental figure S4:**

**
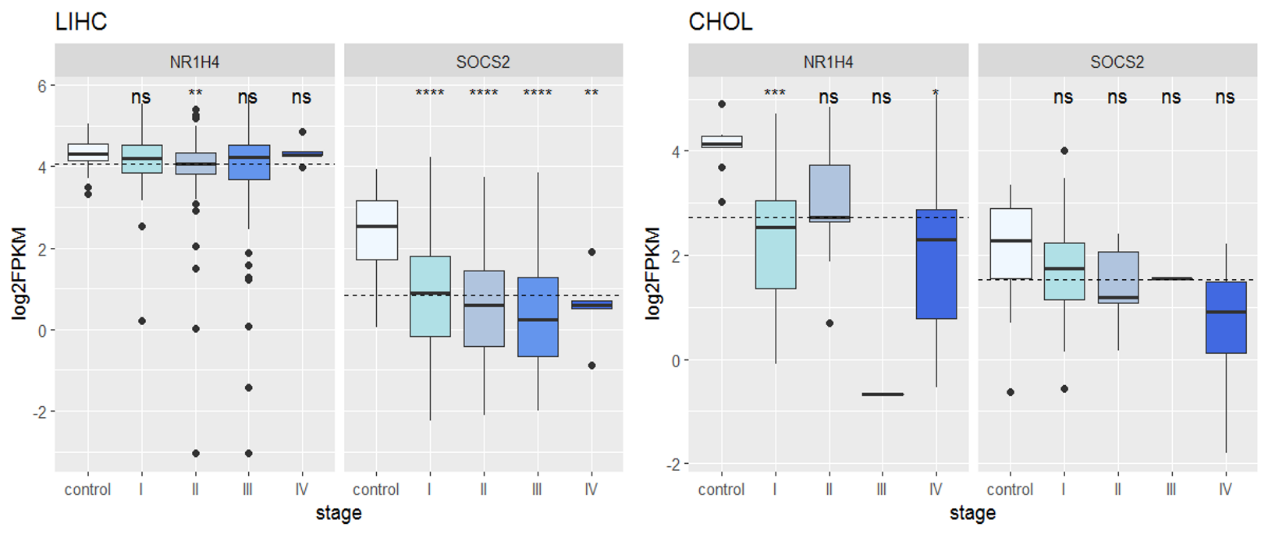
**

**Supplementary Figure S4.** **Clinical relevance of NR1H4 and SOCS2 expression in LIHC and CHOL patients.** Box plot of NR1H4 and SOCS2 expression levels in different phases patients. Statistical significance is determined by 1-way ANOVA multiple comparisons test. **** P < 0.0001, ** P < 0.01, ns P< 1.

**Supplemental figure S5:**

**
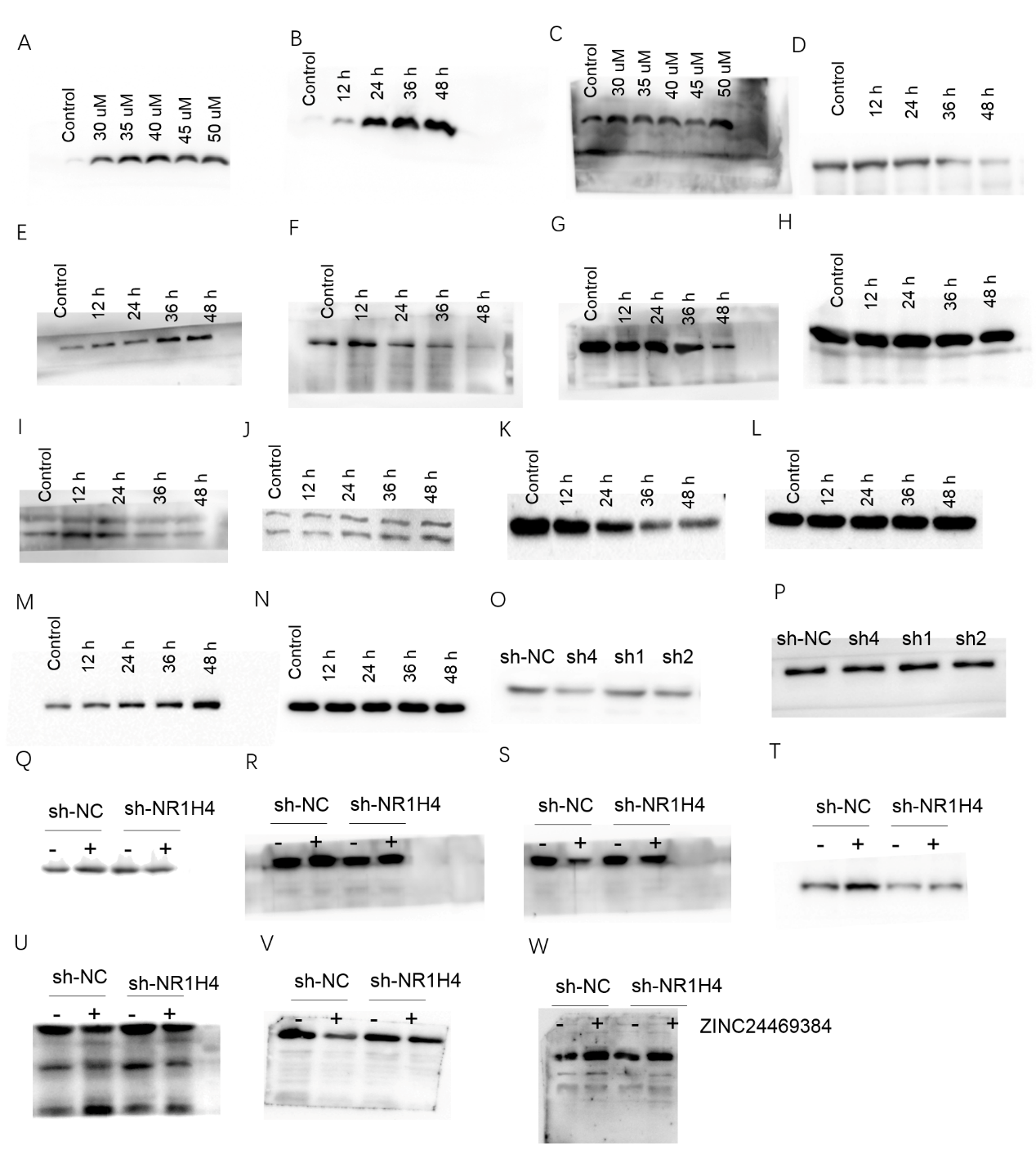
**

**Supplementary Figure S5. Original Western blot images. (A-C) For Figure 2A-B.** A, B anti-Ac-H3; C, anti-H3. **(D-H)** **For Figure 2D.** D, anti-Bcl-2; E, anti-Bax; F, anti-Cyclin A; G, anti-Cyclin B; H, anti-GAPDH. **(I, N) For Figure 6C.** I, anti-Cleaved Caspase 3; J, anti-Cleaved Caspase 9; K, anti-p-STAT3; L, anti-STAT3; M,anti-NR1H4; N,anti-GAPDH. **(O-P)** **For Figure 7A.** O, anti-NR1H4; P, anti-GAPDH. **(Q-W) For Figure 7C.** Q, anti-GAPDH; R, anti-STAT3; S, anti-p-STAT3; T, anti-NR1H4; U, anti-Cyclin A; V, anti-Cyclin B; W, anti-Caspase 3.
